# A preliminary investigation of the subcutaneous tissue reaction to a 3D printed polydioxanone device in horses

**DOI:** 10.1186/s13028-023-00710-0

**Published:** 2023-11-20

**Authors:** Ida Sjöberg, Ellen Law, Fredrik Södersten, Odd Viking Höglund, Ove Wattle

**Affiliations:** 1 Department of Clinical Sciences, Faculty of Veterinary Medicine and Animal Science, Swedish University of Agricultural Sciences (SLU), Box 7054, Uppsala, S-750 07 Sweden; 2 Diagnostic Imaging Clinic, University Animal Hospital, SLU, Uppsala, Sweden; 3 Department of Biomedical Sciences and Veterinary Public Health, Faculty of Veterinary Medicine and Animal Science, SLU, Uppsala, Sweden

**Keywords:** Additive manufacturing, Equine, Ligation, Resorbable device, Tissue reaction

## Abstract

**Background:**

A 3D printed self-locking device made of polydioxanone (PDO) was developed to facilitate a standardized ligation technique. The subcutaneous tissue reaction to the device was evaluated after implantation in ten horses of mixed age, sex and breed and compared to loops of poly(lactic-co-glycolic acid) (PLGA). In two of the horses, the implants were removed before closing the skin. The appearance of the implants and surrounding tissue was followed over time using ultrasonography. Implants were removed after 10 and 27 (± 1) days for histologic examination.

**Results:**

On macroscopic inspection at day 10, the PDO-device was fragmented and the surrounding tissue was oedematous. On ultrasonographic examination, the device was seen as a hyperechoic structure with strong acoustic shadowing that could be detected 4 months post-implantation, but not at 7 months. Histology revealed a transient granulomatous inflammation, i.e., a foreign body reaction, which surrounded both PDO and PLGA implants. The type and intensity of the inflammation varied between individuals and tissue category.

**Conclusions:**

The 3D printed PDO-device caused a transient inflammatory reaction in the subcutaneous tissue and complete resorption occurred between 4 and 7 months. Considering the intended use as a ligation device the early fragmentation warrants further adjustments of both material and the 3D printing process before the device can be used in a clinical setting.

## Background

Biodegradable synthetic polymers have been used in medical devices since the 1980s in both human and veterinary medicine. One of the major advantages with a resorbable device over a non-resorbable device is that it does not require a second surgery. However, it is essential that the device provides sufficient mechanical support during the tissue regeneration process and eventually degrades to non-toxic products with little or no harm to the body. Foreign materials implanted in tissue will trigger an inflammatory response. The type and extent of the response is influenced by the properties of the material and the tissue [1–3] as well as the dimensions, shape and surface characteristics of the material [4–7]. A transient foreign body reaction has been seen with resorbable polymers, but after complete resorption of the materials only scar tissue remains [8–10]. Polydioxanone (PDO) is a resorbable polymer commonly used in equine surgery [11–13]. The polymer is biodegradable and biocompatible and has good flexibility [14], leading to favourable handling characteristics. Although biodegradable polymers are continuously investigated, research is almost exclusively in laboratory animals, aiming towards the use in humans [15–19]. In horses however, there are only a few published studies investigating the subcutaneous tissue reaction to implanted resorbable polymers [20, 21].

Self-locking, non-resorbable, polyamide cable-ties have been used in different surgical procedures, i.e. ligation of the spermatic cords in dogs [22], ovariectomy [23] and ligation of mesenteric vessels [24] in horses. Such self-locking devices are easy and quick to apply and can be tightened around the desired tissue, even when access is limited. However, when leaving non-resorbable material in situ, there is a long term risk of persisting granulomas, adhesions, fistulas and even rejection of the implant [23, 25, 26]. Promisingly, it has been demonstrated that an injection moulded self-locking device, made of resorbable polymers, had adequate clinical performance for ligation in dogs [27, 28] and pigs [29]. This device, made of polydioxanone, has been used when castrating stallions with cryptorchidism laparoscopically [30]. A similar device capable of ligating larger vessels or vascular tissue could be a valuable aid when performing standing castrations in horses. However, the manufacturing process of injection moulding is not flexible with respect to the possibility of modifying the design of the device.

Additive manufacturing, i.e., 3D printing, is a technology that enables the manufacturing of complex structures using a layer-by-layer approach, and it is believed to revolutionize industrial production by decentralising it [31]. The possibility to 3D print resorbable polymers has opened up a wide range of opportunities that, with high flexibility, could customize the design for different applications and transform digital models into unique and on-demand surgical devices. The 3D printing of PDO with the fused filament fabrication (FFF) technology has been successfully demonstrated, and its physical, mechanical and morphological properties were studied before and after degradation in vitro [14, 32]. A scaffold of 3D printed PDO has also been implanted in the distal femur of rabbits with good results [33]. Recently, a 3D printed PDO-device designed for standardized ligation of spermatic cord in horses was developed with features similar to a cable tie. The degradation profile and the mechanical properties of this device have been evaluated in vivo and in vitro [34], but in vivo studies regarding the tissue reactions to 3D printed PDO are still limited.

The objective of the study was to investigate the subcutaneous tissue reaction in horses to a 3D printed self-locking device made of PDO. We hypothesized that the tissue reaction would be similar to that of a traditional resorbable suture material made of a poly(lactid-co-glycolic acid) (PLGA).

## Methods

### Animals

Ten clinically healthy horses (seven Standardbred trotters, one Warmblood and two Shetland ponies) of mixed sex and mean age of 15.4 years (range 9–22) were used in the trial (Table 1). The horses were part of the teaching heard at the Swedish University of Agricultural Sciences and were housed in individual stalls and turned out for < 8 h during the day in a 20 m × 20 m paddock. They were fed haylage and water was available *ad libitum* in both stall and paddock.

**Table 1:** Characteristics of the 10 horses and the randomised location of the implanted materials

Horse	Sex	Age (y)	Breed	Loc 1	Loc 2	Loc 3	Loc 4	Loc 5a	Loc 5b
#1	M	9	STB	PLGA	PDO	PDO	PLGA		
#2	M	9	STB	PDO	PLGA	PLGA	PDO	PDO	
#3	M	17	STB	PDO	PLGA	PLGA	PDO	PDO	
#4	M	10	SWB	PLGA	PDO	PDO	PLGA	PDO	
#5	G	12	STB	PDO	PLGA	PLGA	PDO	PDO	
#6	G	15	STB	PLGA	PDO	PDO	PLGA	PDO	
#7	G	21	SP	PLGA	PDO	PDO	PLGA		PDO
#8	G	22	SP	PLGA	PDO	PDO	PLGA		PDO
#9	M	19	STB	C					
#10	M	20	STB	C					

C, Control; G, gelding; Loc, location; M, mare; PDO, Polydioxanone; PLGA, Poly(lactid-co-glycolic acid); SP, Shetland pony; STB, Standardbred trotter; SWB, Swedish Warmblood; y, years

This study was conducted in accordance with an ethical approval from the Uppsala Animal Ethics Committee. Six of the horses were also included in a concurrent research study investigating recovery from general anaesthesia in healthy horses.

### Materials

**Fig. 1:**
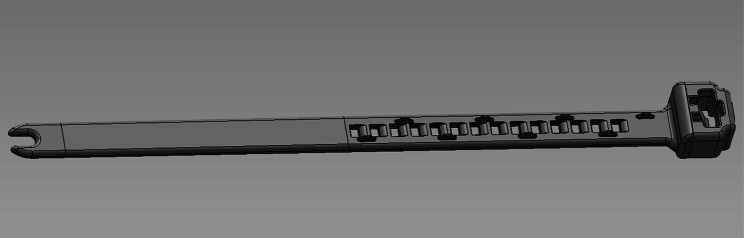
A schematic drawing of the 3D printed device. The device consists of a locking head connected to a partly perforated flexible band

The PDO-device (Dioxaprene, Poly-Med Inc., SC, USA) (Fig. 1) was designed as a flexible, partly perforated band (84 × 4.5 × 0.9 mm) connected to a locking case (6.4 × 8.0 × 4.2 mm) and 3D printed according to the parameters in Table 2. The device, packed in breathable bags, was sterilised with ethylene oxide and dried under vacuum for at least 7 days, then placed inside sealed aluminium foil pouches. As a comparison, we used 20 cm of PLGA (Polysorb USP 2, Covidien, Medtronic, CT, USA). This amount of material corresponds to the amount normally used for ligating spermatic cords when castrating stallions at our university animal hospital.

**Table 2:** 3D printing parameters of the polydioxanone device adapted by Poly-Med

3D printing parameters	
Filament material	Dioxaprene100M
Printing temperature	165 °C
Fan cooling	80%
Extrusion width	0.3 mm
Nozzle size	0.25 mm
Infill percentage	100%
Infill angle	0; 90 °
Infill pattern	Rectilinear
Shell/perimeter	2
Layer height	0.15 mm
First layer height	0.15 mm
Bottom solid layer	0
Top solid layer	1

### Surgical procedure

The surgical procedures were performed by the first author (IS), either under general anaesthesia (n = 6) according to a standard protocol [35], or under routine sedation (n = 4) [36]. For the sedated horses, local infiltration anaesthesia with mepivacaine 20 mg/mL was administrated as an inverted L-block at least 10 cm from the skin incision.

**Fig. 2:**
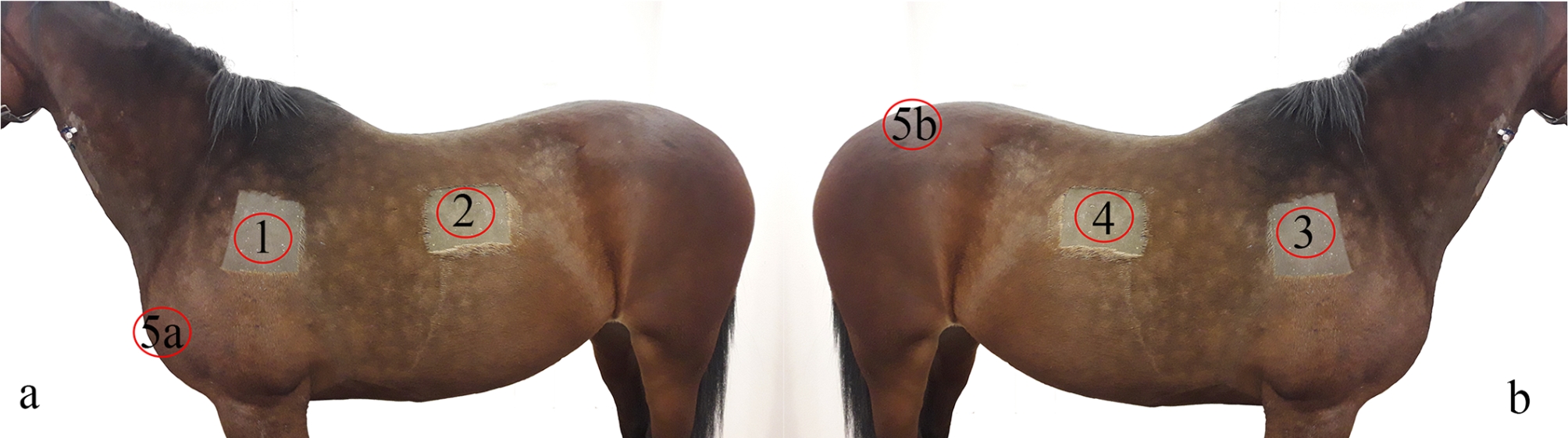
Surgical areas illustrated and location 1–5 denoted with a red circle where implants were inserted subcutaneously in the horse. **a** Left side showing location 1, 2 and 5a. **b** Right side with location 2, 4 and 5b

**Fig. 3:**
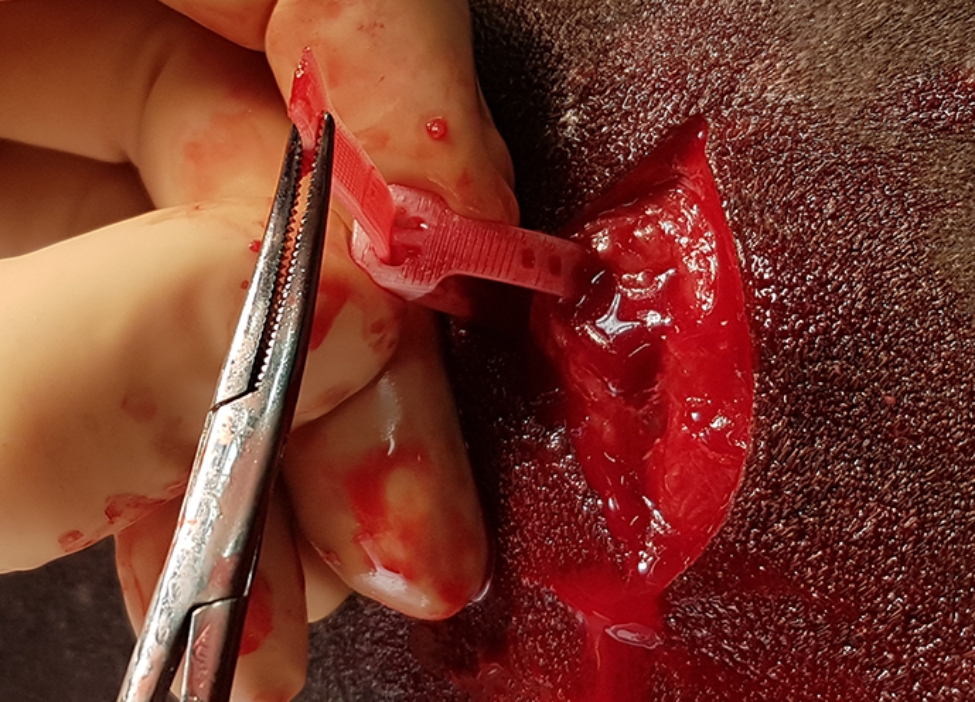
The 3D printed device encircling a piece of fascia, with the free end passed through the locking head

The surgical areas, location 1–5 (Fig. 2), were clipped and aseptically prepared with chlorhexidine soap and ethanol. Via an approximately 8 cm skin incision, a subcutaneous pocket was created in a cranio-dorsal direction with haemostatic forceps. Within the pocket, and at least 5 cm from the incision an implant was attached as follows: The tip of the flexible PDO-band was introduced through the locking case and a self-locking loop was formed encircling subcutaneous tissue (Fig. 3). The loop was tightened and approximately 40 mm flexible band protruding from the locking case was cut off. A loop of double stranded PLGA was placed in a similar way and tied with a surgeon´s knot. The skin was closed with USP 2−0 polypropylene sutures. In the first horse, which served as a pilot, this procedure was repeated in four locations on the left and right side of the trunk, implanting either a PDO-device or a PLGA loop in random order (Table 1). In Horse #2–8, an additional PDO-device, intended for long-time follow up with ultrasound, was implanted at location 5a or b (Fig. 2). In Horse #9 and #10, an incision was made at location 1 as described above, but before closing the skin, the implant was removed. Thereby sham sites where created to serve as controls for inflammation caused by the surgical procedure.

The wounds were protected with a sterile gauze and adhesive foam for at least 48 h post-operatively. The horses were treated with meloxicam 0.6 mg/kg SID IV on the day of surgery and *per os* for three consecutive days. They were monitored daily, with special attention given to signs of inflammation at the surgical areas, until skin sutures were removed 10–14 days after surgery and thereafter in conjunction with ultrasound examinations.

### Ultrasonography

Following routine preparation, i.e. clipping and applying alcohol and/or ultrasound gel, all surgical sites in Horse #1–8 were scanned by a resident in veterinary diagnostic imaging (EL), using a LOGIQ e (General Electrics, Sweden) ultrasound machine with a variable frequency 8–12 MHz linear transducer. According to a set protocol, ultrasound examinations were performed pre-surgery to rule out any pre-existing abnormalities and at 2, 7 and 14 days, 3 and 4 weeks and at 2, 3 and 4 months post-surgery. The areas were examined for remnants of the implants, which were represented by a hyperechoic structure with or without acoustic shadowing, fluid collection, tissue reaction (i.e., hyperechoic tissue) and neovascularization. The implants and surrounding tissues were evaluated transversely, with the probe in a vertical dorso-ventral orientation, and longitudinally, with the probe in a horizontal cranio-caudal orientation. Both static and cine loop B-mode and colour Doppler ultrasound images (gain 20, scale 3 to -3 cm/s) were saved for analysis and evaluated independently by two of the authors (EL and IS).

Since some implants were removed for histologic examination, there were only eight PDO-devices and eight PLGA loops left in situ 4 weeks post-implantation. If the implant was still visible at 4 months, additional examinations were performed at 7 and 8 months post-implantation.

### Sample collection

**Fig. 4:**
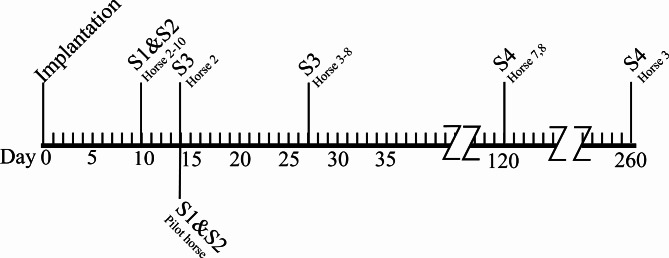
Timeline displaying the days post implantation when samples (S) were excised for histology. S1 Poly(lactid-co-glycolic acid); S2-4 Polydioxanone. The pilot horse was sampled at one occasion, Horses #2, 4–6, 9 and 10 at two, and Horses #3, 7 and 8 at three occasions

Samples (S) of the implants and surrounding tissue were harvested *en bloc* via a 3 cm skin incision over the implant site, identified by palpation or with ultrasound when the implant was not possible to palpate. The implant and approximately 1 cm of surrounding tissue were excised sharply with a scalpel blade and Mayo scissor. In the pilot horse (Horse #1) two samples, one PLGA and one PDO (S1 and S2) were excised at 14 days post-implantation (d14) and were macroscopically inspected. Due to these results, S1 and S2 was excised at d10 (± 1) in the following seven horses. A third sample (S3) of the PDO implant was excised at d14 in Horse #2 and at d27 (± 1) in the remaining six horses. In Horse #9 and #10, subcutaneous tissue from the sham site was excised in a similar fashion as for the implants at d10. The two ponies (Horses #7 and #8) were not going to be accessible after 4 months, which necessitated the collection of additional samples (S4) from location 5b at d120 removing the remains of the PDO-device (Fig. 4). All samples were excised under sedation and local anaesthesia or under general anaesthesia according to the previously described protocols. After gross inspection, the implants and the surrounding tissues were fixated in 4% formaldehyde (Unimedic Pharma AB, Solna, Sweden) and prepared for histology according to routine protocol.

### Histology

Paraffin embedded sections, 4 μm thick and stained with haematoxylin and eosin, were evaluated with light microscopy (Nikon Eclipse 80i, BergmanLabora AB, Danderyd, Sweden) by a pathologist. The tissue inflammatory reaction (TIR) immediately surrounding the implant was assessed by the density of three different types of cells (lymphocytes, macrophages and neutrophils) and assigned a numerical grade from 0 to 3 using a modified Ehrlich-Hunt numerical scale (0 – Absence, 1 – Bare Scattering, 2 – Moderate, 3 – Dense Aggregation). The size of inflammatory zone (IZ) surrounding the implant was semi-quantified using the lowest magnification (4x objective).

### Statistical analysis

Data were analysed in JMP Pro version 16.0.0 (SAS Institute, NC, USA) and a Kaplan-Meier survival graph was used to extract the median time to event for the ultrasound findings. Descriptive data were presented for TIR and IZ as median and interquartile range (IQR).

## Results

### Surgical procedure

The implants were inserted subcutaneously as planned (Figs. 2 and 3), but the tip of the flexible band had expanded during the 3D printing process and needed trimming before introduction into the locking case. Recovery from sedation, anaesthesia and surgery was uneventful and all wounds healed within 10–14 days without intervention or any clinical signs of excessive inflammation.

When the PDO-implant from the pilot horse was harvested for histologic preparation at d14, the device was fragmented and the surrounding tissue was oedematous. Macroscopic inspection of the PDO-implants removed at d10 and d14 in Horse #2 had a similar appearance, therefore samples of implants and surrounding tissue were excised at d10 in the following six horses (Fig. 4). Meanwhile, the PLGA loop was intact and the tissue was denser and integrated with the material. After 27 days, the PDO-device was even further fragmented making it difficult to harvest the biopsy *en bloc*.

### Ultrasonography

**Fig. 5:**
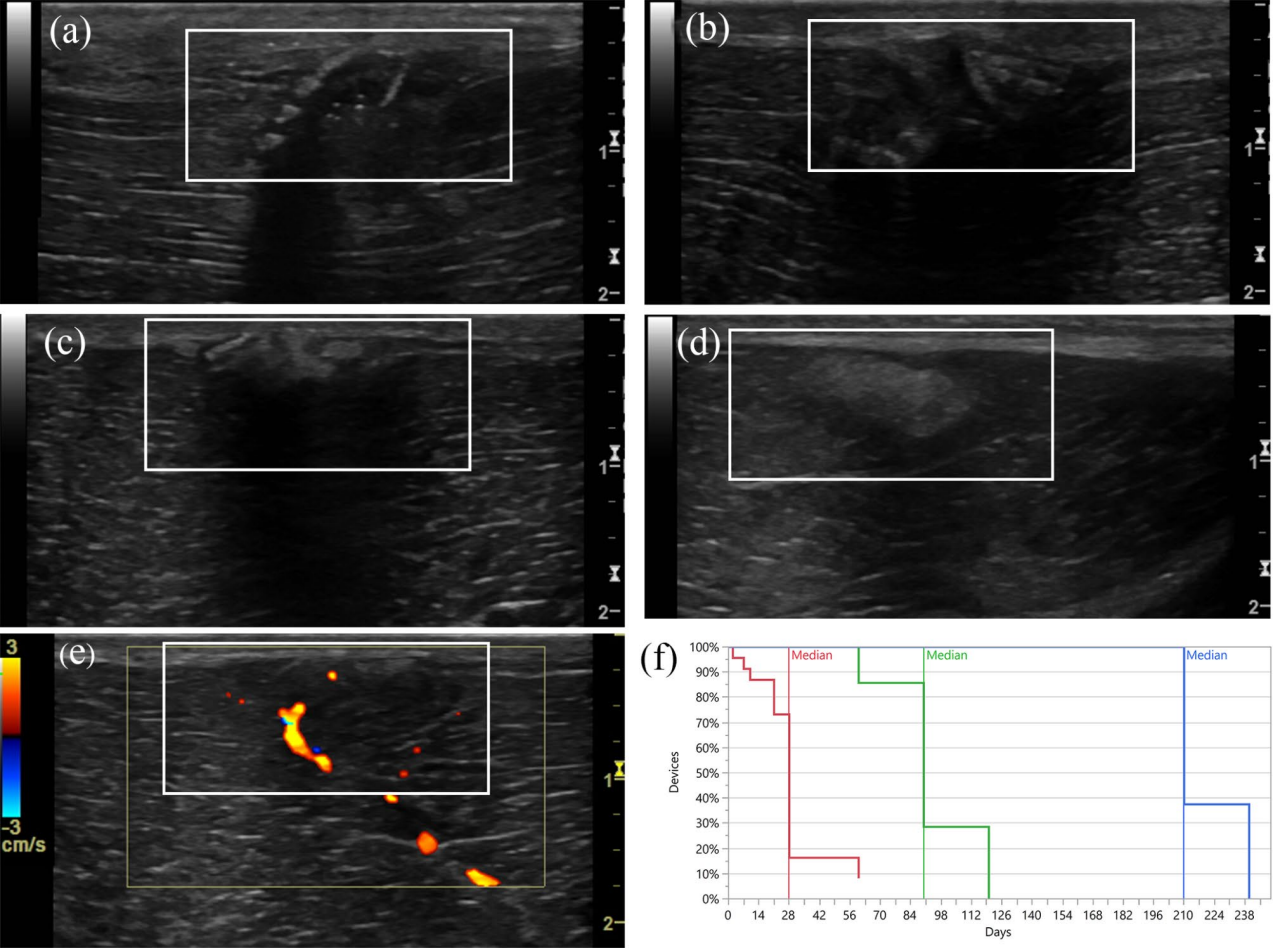
Images depicting ultrasonographic changes of the polydioxanone (PDO) device over time and a graph illustrating the median time of the events. **a** At day 2 the locking case and loop of the device was seen as a hyperechoic ring-like structure with a tail, causing strong acoustic shadowing. **b** At day 28 the device had an amorphous, fragmented look. **c** After day 90 most devices had a rounded homogenous, mildly hyperechoic shadowing appearance. **d** A rounded, smoothly outlined structure was still seen in Horse #3 after 7 months. **e** In Horse #6, neovascularization was observed with colour Doppler in the region of the device at day 90. **f** Kaplan-Meier Survival Plot describing the median time when the PDO-device was fragmented (red), had lost its original shape (green) and was no longer visible with ultrasound (blue)

Over time a range of changes, “events”, were seen on ultrasonographic examination of the 3D printed device. The median time when these events were first registered were extracted from a survival plot (Fig. 5). During the first week, the locking case and loop of the PDO-device were seen as hyperechoic structures with strong acoustic shadowing. Within 2 months, the device gradually obtained an amorphous or fragmented appearance with less acoustic shadowing. After 3 months, the device was seen as an oval shaped, smoothly outlined and well defined structure. At 7 months post-implantation, the tissue had regained its pre-surgical appearance in all horses except one, where a rounded, mildly shadowing, hyperechoic structure could still be seen (Fig. 5d).

During the first 3 weeks, the PLGA-implants were seen as two hyperechoic focal regions, or as thin hyperechoic lines with strong acoustic shadowing. At 4 weeks, the remnants of the implants were difficult to distinguish from surrounding tissues and at 2 months post-implantation, the tissue had regained its pre-surgical appearance.

Regardless of the type of implant, a separation between the subcutaneous tissue and muscle fascial planes was seen at d2. The separation was initially filled with homogenously hypoechoic tissue, which subsequently increased in echogenicity during the following 4 weeks. Neovascularization surrounding the implants was observed in three of the eight horses at different time spans, ranging from d7 to 7 months (Fig. 5e). In Horse #6, this occurred at d7 in locations 3–5a (Fig. 2), but at 3 weeks a vessel was only seen in location 5a, where it persisted for the remaining examination period. In Horses #2 and #4, a small vessel was seen growing into the implant at location 5a, which was visible between 3 weeks and 3 months and from 3 months and onward respectively.

Due to practical reasons, ultrasonography was not performed at d2 in Horses #2, #7 and #8. Since the PDO-implant was fragmented when it was removed for histologic evaluation at d14 in Horse #1, the ultrasound protocol was preponed from d14 to d10 for the remaining horses.

### Histology

**Fig. 6:**
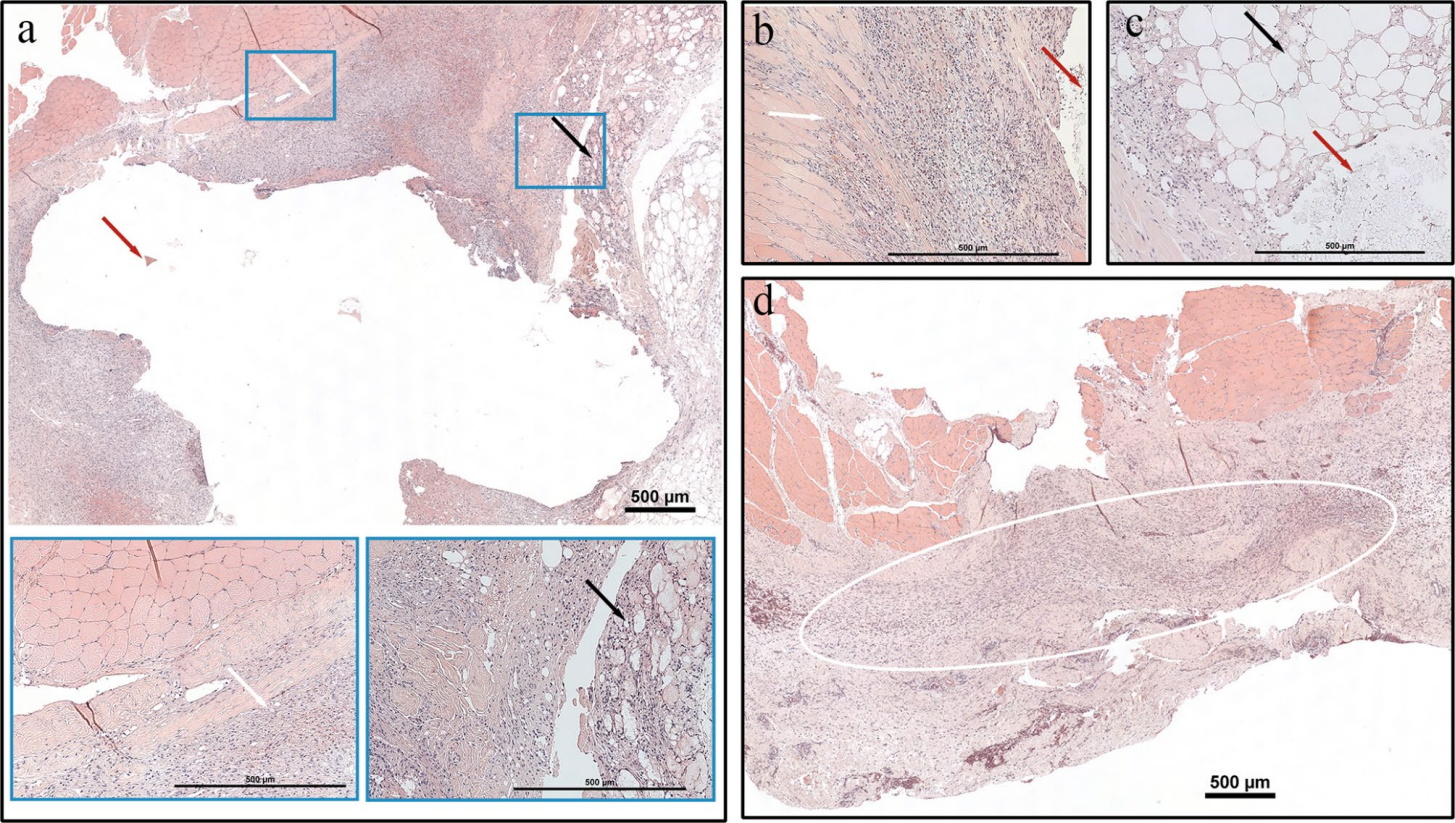
Photomicrographs with haematoxylin and eosin staining that show implanted material or its lumen (red arrows) and surrounding tissue reaction at day 10. **a** Polydioxanone device magnified x4 in the top frame and x20 in the 2 bottom frames. **b, c** Poly(lactid-co-glycolic acid)magnified x20. **d** Control wound magnified x4. Surrounding the implanted material a more restricted inflammation was seen in areas adjacent to the muscle tissue (white arrows), whereas there was a more prominent inflammation in the adipose tissue with steatitis (black arrows). In the control wound, there was a mild chronic inflammatory reaction (white oval). Bar scale = 500 μm

**Fig. 7:**
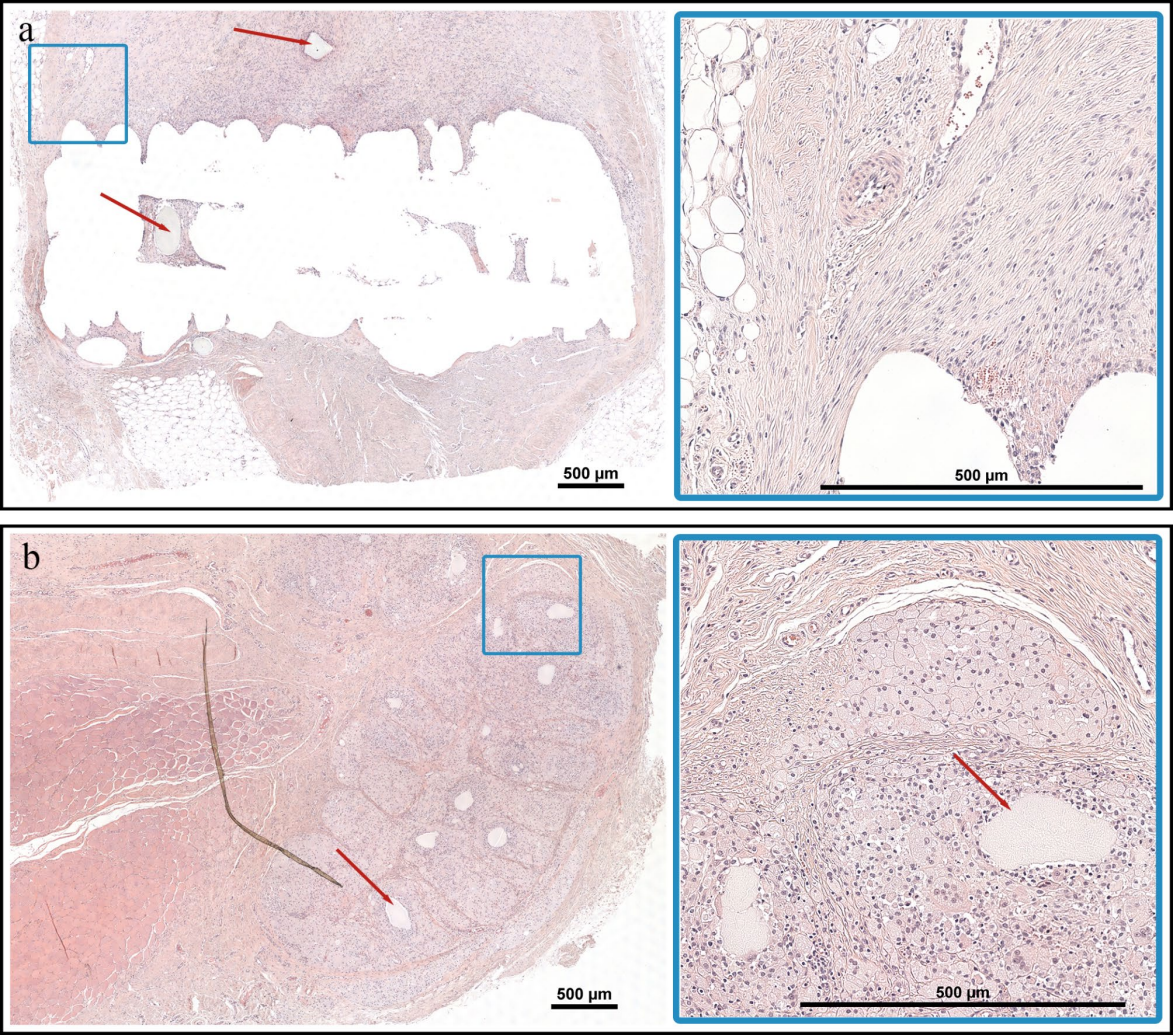
Photomicrographs with haematoxylin and eosin staining of the progressing tissue reaction to the polydioxanone (PDO) device (red arrow). **a** Sample collected at day 27. **b** Sample collected at day 120. Blue square to the right magnified x20. Over time, the inflammation around the PDO-device became more mature and organized with decreasing numbers of inflammatory cells and a more compact, and less cellular, connective tissue. At day 120 there was granulation tissue encapsulating the remnants of the PDO-device. Bar scale = 500 μm

The inflammatory reactions in the tissue surrounding both the implants were characterized by chronic granulomatous inflammation containing elements of foreign body reaction, mainly concentrated around the implants. The inflammation was dominated by macrophages mixed with a variable ratio of neutrophils ranging from 5 to 40%. In one horse (#6), an eosinophilic response surrounded the PLGA implant. The TIR grades and the proportion of the IZ at d10 and d27 are presented in Table 3. The type and intensity of the inflammation varied between individuals and different categories of tissue, regardless type of implant. The implants adjacent to adipose tissue showed a more prominent inflammation with degeneration and necrosis of the adipocytes, whereas in areas of muscle tissue, the inflammatory response was less abundant (Fig. 6a-c). In the two control wounds, there was a mild chronic inflammatory reaction with areas of fibrotic tissue and neovascularization (Fig. 6d). As time progressed, the inflammatory reaction matured and was subjectively more organized (Fig. 7a). In a majority of horses, macrophages represented ≥ 90% of the inflammatory cells at d27. However, in one horse (#8) a 20% infiltration of lymphocytes was present. After 4 months, the signs of chronic inflammation were replaced by granulation tissue encapsulating the remnants of the PDO-device (Fig. 7b). In Horse #3, where a persistent hyperechoic region was seen with ultrasound, a tissue sample of the region at d260 revealed a thin band of fibrotic tissue without any inflammatory component or remnants of the device.

**Table 3:** Tissue inflammatory reaction (TIR) and inflammatory zone (IZ) around the implants

	TIR (grade)			IZ (%)		
	d10		d27	d10		d27
Horse	PLGA	PDO	PDO	PLGA	PDO	PDO
**#2**	1	2		50	40	
**#3**	1	3	1	15	25	10
**#4**	2	1	1	100	15	15
**#5**	2	3	1	85	85	10
**#6**	2	2	1	60^*^	20	10
**#7**	1	2	2	30	90	15
**#8**	2	2	1	50	50	35^¤^
**Median**(IQR)	**2** (1–2)	**2** (2–3)	**1** (1–1)	**50** (30–85)	**40** (20–85)	**12,5** (10–20)

The tissue inflammatory reaction in samples collected at day (d) 10 and 27 was assessed with histology using a modified Ehrlich-Hunt numerical scale from 0–3. The inflammatory zone was semi-quantified as a proportion of the total field view using the lowest magnification (4x objective)

* 4% of the inflammatory reaction were represented of eosinophils

¤ 20% of the inflammatory reaction were represented of lymphocytes

IQR, Inter quartile range; PDO, Polydioxanone; PLGA, Poly(lactid-co-glycolic acid)

## Discussion

The 3D printed PDO-device did not cause any clinical signs of inflammation in the subcutaneous tissue, although complete resorption was not seen prior to 7 months post-implantation. The device however, was already fragmented 10 days after implantation, which suggests that its mechanical properties decreased faster than anticipated. On histologic examination, there was a transient foreign body reaction, but with great variation between the different regions within one sample.

Ultrasonography was used to follow the appearance of the implants and surrounding tissue reaction over time. Initially both the PDO-device and the PLGA loop were seen as hyperechoic structures with strong acoustic shadowing. The hypoechoic tissue separation surrounding the implants was likely oedema or seroma formation related to the surgical procedure, and the space was filled with granulation tissue within 4 weeks. This corroborates the results presented in a similar study of resorbable polymers implanted in horses [20]. All but one PDO-device had an intact appearance on ultrasound during the initial 4 weeks. As the devices exhibited strong shadowing properties, and some parts of the device often shadowed other parts, a full evaluation of the entire device was not always possible. This could be the reason why fragmentation was not observed on ultrasonography earlier. In contrast to the findings from Höglund et al. [37], all remaining devices were still visible 4 months post-implantation. A plausible explanation could be that the devices in the current study were larger, which resulted in a greater amount of material to be absorbed, or access for imaging was easier since the devices were implanted subcutaneously. In regard to factors such as vascularization and transport capacity, it should be noted that comparisons with previous studies must always be made cautiously as there are differences between the tissues and species studied [2, 38]. Ideally, additional ultrasound examinations should have been performed at 5 and 6 months to better pinpoint the exact time when the device was no longer visible. This was not possible since the horses used in this study were not accessible during this period. On the other hand, the exact resorption time of a material is difficult to assess with ultrasound [20, 37]. This difficulty was further demonstrated in this study where remnants of the device could not be found on histologic examination, even though the preceding ultrasound images showed a hyperechoic structure with mild acoustic shadowing.

Histologically both materials caused low-grade inflammatory reactions which were confined to the surrounding tissue, which is in agreement with previous studies [18, 37, 39]. The magnitude of the reaction was highly related to the type of surrounding tissue, where materials implanted adjacent to subcutaneous fat resulted in a more prominent reaction, than tissue consisting of muscle fibres. This was not anticipated, but likely a spill over effect initiated by the handling of tissue during surgery, as adipose tissue is more prone to undergo degeneration and necrosis than muscle fibres. If clearance of polymeric hydrolytic debris is less in fat than muscle, then that will also contribute to a greater tissue reaction [2, 3]. In Horses #7 and #8, the remains of the PDO-devices were excised at 4 months, giving us additional information on the resorption progress of the device. This further demonstrated the transient inflammatory response and was in agreement with the findings from Carvalho et al. [21], where another type of polymer was implanted in a horse. PLGA was used as a control material since it is commonly used for ligating spermatic cords in equines. It has favourable handling characteristics and a limited, transient tissue reaction in laboratory animals [39]. The histological findings were in agreement with previous studies [39, 40], although one horse had an eosinophilic inflammatory response around the PLGA implant, which could be a normal individual variation.

Implants 3D printed with the FFF technique will expand while cooling down on the printer bed. The material was processed through a heated printer nozzle and deposited onto molten layers. The produced volume may, to some extent, “flow out” beyond its intended shape, which probably caused the difficulty in fitting the tip into the locking case. Improving the original design by tapering the tip could be a simple adjustment using 3D printing technology.

The early fragmentation of the device was unexpected and not in accordance with some previous in vitro [19, 41] and in vivo [16, 19] studies of the same material. However, it has been shown that polydioxanone fibres degraded significantly faster in living tissue than in saline solution [7]. When Adolfsson et al. [34] compared in vitro and in vivo degradation of the tested device, it was found that in vivo aging had a completely different impact on the mechanical behaviour of the devices, which resulted in an earlier and more severe loss of tensile strength and decreased elasticity. The authors suggested that the 3D printing process and anatomic location could influence the degradation process, and highlighted the importance of the in vivo testing of a device before clinical application.

### Study limitations

Firstly, the creation of a surgical wound triggers a tissue response, which may interfere with the response to the implanted material. In this study, an effort was made to limit this interference by positioning the materials away from the skin incision, and by comparing the results to sham wounds. Additionally, the aim was to evaluate a device intended to be used in a surgical environment. Secondly, the tested device is proposed to be used for ligation of the spermatic cord when castrating horses standing. However, this area is difficult to access and requires that the horse is a stallion, why the device was implanted subcutaneously on the trunk of the horses instead. Resorption time may differ between different anatomical locations [2], an important discrepancy that always needs to be acknowledged when using implants. Thirdly, the tissue reaction varied greatly between different regions within one sample, which was not expected and made the histologic scoring difficult. Scoring tissue reaction is challenging, as there are always elements of subjectivity and to the authors’ knowledge, there is no gold standard protocol. The histological examination could not be blinded since remnants of the implants could be seen in the sections. The large individual variation resulted in low statistical power, which is why descriptive data was presented.

## Conclusions

The 3D printed PDO-device only caused a transient foreign body reaction in the subcutaneous tissue, similar to that of PLGA. Complete resorption of the device occurred between 4 and 7 months, but function was lost already before 10 days post-implantation. Continued adjustments of the design of the device, polymer material and the 3D printing process is warranted before the device can be used in a clinical setting. A different resorbable polymer or a PDO composite aimed to strengthen the device and prevent early fragmentation might be a solution in the quest for a functional device. This study also reveals the limitations of ultrasonographic examination when it comes to evaluating the resorption process of implants and the surrounding tissue reaction.

## Data Availability

The datasets generated during and/or analysed during the current study are available from the corresponding author on reasonable request.

## Declarations

### Prior publication

Some of the data included in this article have previously been published as an abstract in the Proceedings of the ACVS Surgery Summit 2020, Virtual Session, DOI: 10.1111/vsu.13506.

## Abbreviations

FFF: Fused filament fabrication
IZ: Inflammatory zone
PDO: Polydioxanone
PGLA: Poly(lactid-co-glycolic acid)
S: Sample
TIR: Tissue inflammatory reaction
